# Loss of ADAM9 Leads to Modifications of the Extracellular Matrix Modulating Tumor Growth

**DOI:** 10.3390/biom10091290

**Published:** 2020-09-07

**Authors:** Anna N. Abety, Elke Pach, Nives Giebeler, Julia E. Fromme, Lavakumar Reddy Aramadhaka, Cornelia Mauch, Jay W. Fox, Paola Zigrino

**Affiliations:** 1 Department of Dermatology, University of Cologne, 50937 Cologne, Germany; annaabety@yahoo.com (A.N.A.); elke.pach@uk-koeln.de (E.P.); sevinoro@googlemail.com (N.G.); julia.fromme@uk-koeln.de (J.E.F.); cornelia.mauch@uk-koeln.de (C.M.); 2 Department of Microbiology, Immunology and Cancer Biology, University of Virginia, Charlottesville, VA 22908-734, USA; lavakumarreddya@gmail.com (L.R.A.); jwf8x@virginia.edu (J.W.F.)

**Keywords:** ADAM9, collagen type I, extracellular matrix, fibroblasts, melanoma

## Abstract

ADAM9 is a metalloproteinase strongly expressed at the tumor-stroma border by both tumor and stromal cells. We previously showed that the host deletion of ADAM9 leads to enhanced growth of grafted B16F1 melanoma cells by a mechanism mediated by TIMP1 and the TNF-α/sTNFR1 pathway. This study aimed to dissect the structural modifications in the tumor microenvironment due to the stromal expression of ADAM9 during melanoma progression. We performed proteomic analysis of peritumoral areas of ADAM9 deleted mice and identified the altered expression of several matrix proteins. These include decorin, collagen type XIV, fibronectin, and collagen type I. Analysis of these matrices in the matrix producing cells of the dermis, fibroblasts, showed that ADAM9^−/−^ and wild type fibroblasts synthesize and secreted almost comparable amounts of decorin. Conversely, collagen type I expression was moderately, but not significantly, decreased at the transcriptional level, and the protein increased in ADAM9^−/−^ fibroblast mono- and co-cultures with melanoma media. We show here for the first time that ADAM9 can release a collagen fragment. Still, it is not able to degrade collagen type I. However, the deletion of ADAM9 in fibroblasts resulted in reduced MMP-13 and -14 expression that may account for the reduced processing of collagen type I. Altogether, the data show that the ablation of ADAM9 in the host leads to the altered expression of peritumoral extracellular matrix proteins that generate a more favorable environment for melanoma cell growth. These data underscore the suppressive role of stromal expression of ADAM9 in tumor growth and call for a better understanding of how protease activities function in a cellular context for improved targeting.

## 1. Introduction

The ability of tumor cells to grow and invade the surrounding tissue depends on their ability to remodel the extracellular matrix (ECM) directly or indirectly as a result of cellular communication with stromal cells [1]. Fibroblasts are the primary producers of matrices and produce a variety of proteases involved in remodeling of the ECM, thus modulating its functional characteristics. These proteases include serine-, cysteine-, aspartyl-, and metalloproteases [2]. Among the metalloproteinases there are the matrix modifying enzymes, MMPs (Matrix Metalloproteinases), and the ADAMs (a disintegrin and metalloprotease) proteases. ADAMs are multidomain, type I transmembrane proteins characterized by a conserved domain structure. This includes a prodomain, metalloprotease, disintegrin, cysteine-rich, epidermal growth factor-like, and transmembrane domains, and a cytoplasmic tail [3,4]. ADAM9, a member of the ADAM family, is widely expressed and is up-regulated in several human cancers such as breast, prostate cancer, and melanoma [4,5,6,7,8]. ADAM9 can shed collagen XVIl and process fibronectin, proteins involved in cell-matrix adhesion and migration [9,10]. Studies have shown that ADAM9 regulates cell–cell and cell–matrix interaction by binding to integrins, thereby leading to modulation of protease secretion and cell migration [11,12,13]. ADAM9 expression in melanoma cells contributes to melanoma progression modulating cell adhesion to the endothelium and altering basement membrane (BM) integrity by proteolytically processing the laminin-beta3 chain [14]. On the other hand, ADAM9 is found to be highly expressed in peritumoral areas where activated fibroblasts are found [14,15]. In these cells, altered expression of ADAM9 controls TIMP-1 expression and TNF-α/sTNFR1 pathway leads to modulation of melanoma cells proliferation and apoptosis [15]. An additional described function of ADAM9 in vitro is the processing of proteins of the dermal ECM, for instance, fibronectin [10]. To evaluate the role of ADAM9 in the modification of the ECM during melanoma development, we have analyzed the ECM at the tumor-stroma border in B16F1 melanomas developed in wild type or ADAM9^−/−^ mice. Several ECM proteins are altered in the absence of stromal-derived ADAM9, most prominent is the increased type I collagen. Besides using in vitro cell culture systems and recombinant ADAM9, we addressed the role of fibroblast-derived ADAM9 in the regulation of ECM protein expression and the functional consequences for these on cell growth.

## 2. Materials and Methods

### 2.1. Cell Culture Cells and Reagents, Preparation of Conditioned Media

Murine dermal fibroblasts were isolated from newborn skin of wild type (WT) and ADAM9^−/−^ mice, as previously described [16]. Isolated fibroblasts were used between passages 2 and 4 for experiments. COS-7 cells, murine fibroblasts, and B16F1 murine melanoma cells were cultured in DMEM supplemented with 10% FCS, 2 mM glutamine 100 U/mL penicillin and 100 µg/mL streptomycin. For the preparation of cell culture supernatants, cells were cultured until 90% confluent, washed twice with phosphate-buffered saline (PBS), and incubated overnight with serum-free medium. The supernatant was clarified by centrifuging at 2000× *g* for 5 min and used immediately or stored at −20 °C.

### 2.2. Proliferation Assays

B16F1 cells were seeded in 96-well plates (10^4^ cells/cm^2^) coated with different concentrations of purified acid-soluble rat collagen type I (Cell Systems, Remagen, Germany) diluted in PBS at 4 °C overnight. Cells were cultured in serum-free medium for 24 h. Cell proliferation was assessed using the BrdU Cell Proliferation ELISA kit (Roche Diagnostics, Mannheim, Germany) according to the manufacturer’s instructions.

### 2.3. Preparation of Recombinant ADAM9

The plasmid encoding the soluble ADAM9 was a kind gift of Alex Toker (Beth Israel Deaconess Medical Center, Harvard Medical School, Boston, MA, USA). To introduce the specific mutation of the “catalytic” glutamic acid in the metalloproteinase active site (Glu → Ala; E > A), we used the transformer site-directed mutagenesis kit (Clontech Laboratories Inc., Heidelberg, Germany). This mutation was shown to result in loss of proteolytic activity of ADAM9 towards insulin b chain [17]. The insertion of mutation was verified by DNA sequencing. COS-7 cells were seeded at a density of 1 × 10^3^ cells per cm^2^. At confluency of 90%, cells were transfected with two µg of pcDNA4/To/myc-His containing either ADAM9 soluble or the mutated (E > A) form using Lipofectamine 2000 (Invitrogen, Karlsruhe, Germany). Cells were cultured in DMEM for 24 h and clones selected by culturing in the presence of 500 µg/mL zeocin (Invitrogen). Serum-free cellular supernatants from established clones were collected and clarified by centrifugation at 1000× *g* for 5 min. TALON metal affinity resins (Clontech) were added to the supernatant at a ratio of 1:100 and incubated overnight at 4 °C. Resins were washed twice, and bound proteins were eluted with 250 mM imidazole overnight at 4 °C. Dialyzed concentrated purified proteins were aliquoted and stored at −20 °C for further analysis.

### 2.4. Western Blot Analysis

Conditioned media were concentrated by TCA precipitation and solubilized in Laemmli sample buffer. An equal amount of proteins were separated under reducing conditions on a 10% or 12% polyacrylamide gel. Resolved proteins were transferred onto a Hybond-C nitrocellulose membrane (GE Healthcare, München, Germany) in a semidry transfer system. The transfer efficiency was assessed by staining the membrane with Ponceau Red (Sigma-Aldrich, Diesenhofen, Germany). Membranes were blocked for 1 h with 5% milk powder and incubated overnight at 4 °C with the primary antibody. Primary antibodies used were anti-ADAM9, anti-decorin, (AF949 and MAB143, respectively, all from R&D Systems, Wiesbaden, Germany), anti-collagen type I (1:500; 20315035505 from Quartett, Berlin, Germany), anti-fibronectin and anti-actin (F3648 and A2668, both 1:1000; from Sigma Aldrich, Diesenhofen, Germany), and anti-MMP-13 (sc-30073, from Santa Cruz, Heidelberg, Germany; 1:1000), anti-MMP-14 (1:500) [18]. After washing, membranes were incubated with HRP-labeled secondary antibody (from Dako, Hamburg, Germany) for 1 h at room temperature. Bound secondary antibodies were detected by ECL^®^ system (Thermo Scientific, Bonn, Germany) and the membranes exposed to X-ray films (Thermo Fisher, Karlsruhe, Germany).

### 2.5. Zymographic and In Vitro Enzymatic Assays

Cell culture supernatant was fractionated by SDS-PAGE containing 1 mg/mL bovine gelatin (Sigma Aldrich, Diesenhofen, Germany). After electrophoresis, the gels were washed in 2.5% Triton X-100 for 30 min and incubated overnight in substrate buffer (50 mm Tris-HCl, 5 mm CaCl_2_, pH 8.0). The gels were stained with 0.25% coomassie brilliant blue G250 (Serva, Heidelberg, Germany), bands corresponding to gelatinase activities appeared white against the blue background. For the enzymatic assay, 500 ng of recombinant ECM proteins was incubated with 1µg purified recombinant sADAMS-9 in substrate buffer overnight at 37 °C. The reaction was terminated by heating with 5 µL Laemmli reducing loading buffer at 95 °C for 5 min and analyzed by western blot.

### 2.6. RNA Isolation, RT-PCR and Real-Time PCR

Total RNA from cells was isolated, as previously described [19]. Primers for the amplification of murine MMP-13, MMP-14, and S26 were described elsewhere [19]. For quantitative real-time PCR analysis, reversed transcribed RNA was used for real-time PCR using the StepOne RealTime (Applied Biosystems, Thermo Scientific, Bonn, Germany). Five microliters of sample cDNA were added to the master mix, and the amplification performed in a total volume of 20 µL for 40 cycles. The thermal cycling conditions were set to 50 °C for 2 min and 95 °C for 10 min, followed by 40 cycles of amplification at 95 °C for 15 s and 60 °C for 1 min for each cycle. Primers used for real-time amplification were as follows: decorin sense 5′-TGAGCTTCAACAGCATCACC-3′, antisense 5′-AAGTCATTTTGCCCAACTGC-3′; collagen type I sense 5′-CCCCTGGTCTTACTGGGAAC-3′, antisense 5′-AGCAGGTCCTTGGAAACCTT-3′.

### 2.7. Tumor Growth Assay In Vivo

The in vivo tumor experiments were performed as previously described [15]. Briefly, B16F1-GFP murine melanoma cells (1 × 10^6^ cells) in 0.02 mL phosphate-buffered saline were injected intradermally into the flank of 6- to 8-week old, WT and ADAM9^−/−^ littermate mice. Tumor size was measured using a precision caliper (Mitutoyo, Neuss, Germany) and mice euthanized at day three post-injection. Animal experiments followed the ethical standards approved by the local veterinary authority (NRW authorization 87-51.04.2010.A365).

### 2.8. Mass Spectrometry

Formalin-fixed paraffin-embedded (FFPE) tissue samples from three independent tumors per genotype were sectioned (10 µm sections). We used tumors at three days post B16F1 injection corresponding to the earliest time point at which tumor size between WT and ADAM9^−/−^ was significantly different [15]. Proteins used for mass spectrometry were isolated from the tumor-stroma border by laser dissection using a Leica ASLMD instrument (Leica Microsystems Inc., Buffalo Grove, IL, USA). Processing of tissues and mass analysis was performed as elsewhere described [20]. Values obtained from the study of two mice tissue specimens per genotype were probabilistically validated by Scaffold Software Q+ (4.11.0) [20] and only proteins identified by two or more peptides and a minimum protein identification probability of >95% were listed. The ratios of averaged values were used here.

### 2.9. Histology

Tumors were removed surgically, one half was embedded in optimal-cutting-temperature compound (O.C.T., Vogel, Giessen, Germany) and frozen, the other half was fixed in 4% formalin and embedded in paraffin. For immunofluorescence analysis, 8 µm cryosections were fixed in ice-cold acetone for 8 min and incubated with primary antibodies overnight at 4 °C at a dilution of 1:100. Primary antibodies used were anti-collagen type I (from Abcam, Cambridge, UK), anti-collagen type XIV (generously provided by Prof. Manuel Koch, Institute for Dental Research and Oral Musculoskeletal Biology, University of Cologne), anti-decorin from R&D Systems (Wiesbaden, Germany), anti-fibronectin (from Sigma Aldrich, Diesenhofen, Germany). Bound primary antibodies were detected with goat anti-rat-Alexa 488-conjugated secondary antibodies (Invitrogen, Darmstadt, Germany). Nuclei were stained using 4,6-diamidino-2-phenylinodole (DAPI; 1:1000; Roche Diagnostics, Mannheim, Germany).

### 2.10. Statistical Analysis

All experiments were performed on groups of independently isolated fibroblasts populations for each genotype and repeated as indicated. Statistical analysis of the data was performed using the Student *t*-test. Differences with *p* < 0.05 were considered statistically significant.

## 3. Results

### 3.1. ECM Protein Expression at the Tumor-Stroma Border of B16F1 Melanomas

**Figure 1:**
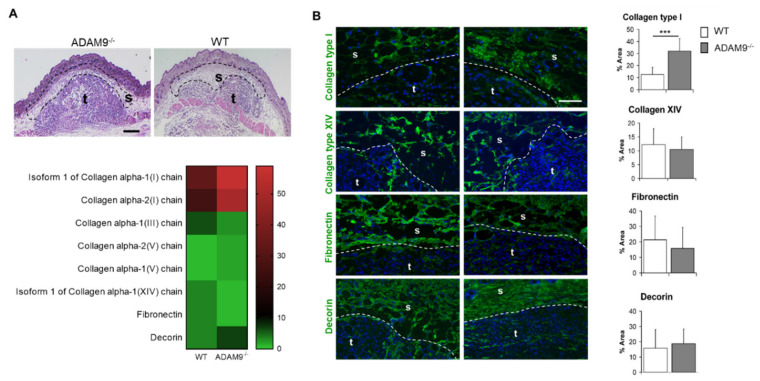
Differential expression of ECM proteins in the absence of ADAM9. (**A**) Analysis of ECM proteins at the tumor-stroma border. Black dotted lines indicate the microdissected areas used for mass spectrometry. t, tumor; s, stroma. Scale bar 50 µm. In the lower heatmap are the relative intensities of protein expression in ADAM9^−/−^ and WT. (**B**) Immunofluorescence analysis of collagen type I, collagen type XIV, decorin, and fibronectin (green, nuclei: blue). As a negative control, sections were incubated in a blocking buffer without the primary antibody. Representative pictures are shown (WT *n* = 5; ADAM9^−/−^ *n* = 5). Scale bar 400 µm. Staining was quantified in five different fields from 3 mice each genotype. Densities are shown as the percentage of the measured area; these are on the right. *** *p* < 0.0005.

By grafting of ADAM9 expressing B16F1 melanoma cells in animals carrying complete ablation of this protease, we could show that in vivo ablation of this protease in stromal fibroblasts leads to enhanced tumor growth as a result of altered cellular activities [15]. In vitro, ADAM9 was shown to process extracellular matrix proteins as fibronectin and laminin [10,21]. Therefore, we sought to analyze whether alterations in extracellular matrix components might have contributed to modulating melanoma cell growth in vivo. Modifications in the ECM were analyzed by mass spectrometry in proteins from the peritumoral stroma of WT or ADAM9^−/−^ mice carrying B16F1 melanomas, as described earlier [15]. The area examined is indicated by the black dotted lines in the HE stained sections (Figure 1A, upper panel). Several proteins were aberrantly regulated, and among these were ECM proteins and other enzymes altered at the tumor-stroma border in the absence of host-derived ADAM9.

Four hundred and ninety-six of the analyzed proteins were similarly expressed in both genotypes, and only 53 proteins were altered at the tumor-stroma border of tumors from ADAM9^−/−^ animals compared to WT controls. Among these, only extracellular matrix proteins (Figure 1A), metabolic proteins, or intracellular enzymes (Supplementary Table S1) with a minimum fold change of at least two were considered relevant. However, most of these enzymes have primary roles in cellular metabolism more than matrix remodeling in the microenvironment. The extracellular matrix proteins included collagen type V, collagen type I, and decorin, which were up-regulated in tumors from knockout animals compared to those from WT. In contrast, collagen type XIV and fibronectin were down-regulated (Figure 1A, lower panel). Interestingly, analysis of healthy skin from both mice genotypes did not display any alterations in collagen type I, fibronectin, and collagen type XIV (Figure S1). Antibodies to collagen type I detected a significant increase of collagen in peritumoral areas juxtaposed to the tumor (Figure 1B). Decorin levels were only modestly increased at the tumor-stroma border in ADAM9^−/−^ mice, while reduced expression of collagen type XIV and fibronectin was detected (Figure 1B). Quantification of positive stain in the tumor-stroma edge corroborated the increased expression of collagen type I but did not indicate regulation of the other analyzed proteins decorin, fibronectin, and collagen type XIV (Figure 1B right panel)

### 3.2. Increased Collagen Type I by Fibroblasts in the Absence of ADAM9

**Figure 2:**
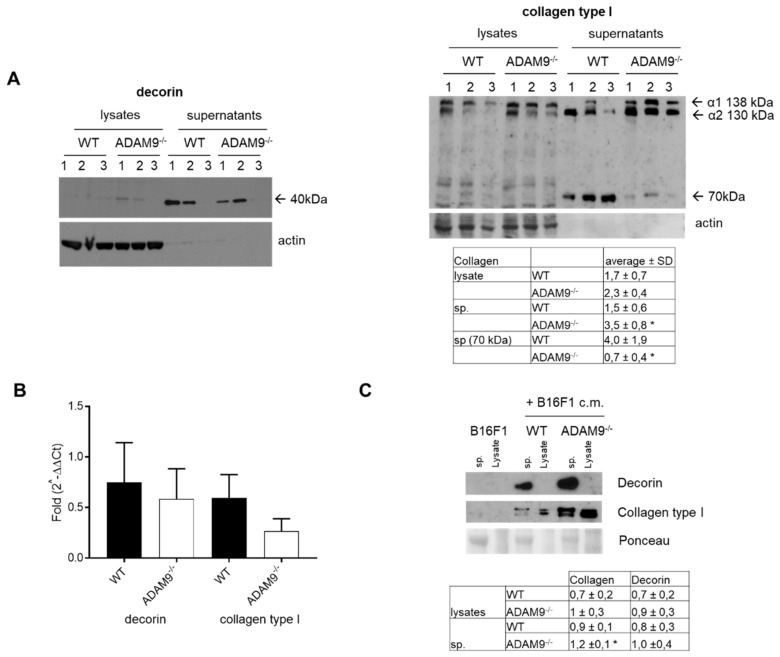
Expression of decorin and collagen type I in ADAM9 deficient fibroblasts. (**A**) Immunoblot analysis of decorin and collagen type I in cell supernatants and lysates from monolayer cultures of ADAM9 deficient or wild type dermal fibroblasts. Actin was used as a control. Underneath, in the table, is the average densitometric analysis after normalization to control (*n* = 3; * <0.03–4) (**B**) Quantitative real-time PCR analysis of decorin and collagen type I was performed using S26 as an internal control for normalization of transcripts. Data are mean values ± SEM (average of 6 different fibroblasts cultures per genotype). (**C**) Immunoblot analysis of decorin and collagen type I in cell supernatants (sp.) and lysates from monolayer cultures of B16F1, ADAM9^−/−^, or wild type dermal fibroblasts, without or with stimulation B16F1 conditioned medium (B16F1 c.m.). Underneath, in the table, is the average densitometric analysis of collagen and decorin after normalization to control (*n* = 4; * <0.01). Ponceau staining of the membrane was used as a control for loading.

Increased synthesis of matrix components by stromal cells has been reported in several tumors, including skin cancer [22,23]. One of the suggested underlying mechanisms leading to the enhanced expression of matrix and its accumulation is the cellular communication of melanoma cells with the surrounding stroma [24]. That may lead to an altered turnover, thus modifications in protein synthesis and secretion, and altered proteolytical processing. To investigate the proteolytical role of fibroblasts-derived ADAM9 to matrix protein processing, we used monolayer cultures of fibroblasts. Synthesis and secretion of decorin and collagen type I was evaluated in control WT and ADAM9 deficient dermal fibroblasts grown as monoculture (Figure 2A). These experiments were performed using three independently isolated fibroblast populations from each genotype to ensure reproducibility. Decorin in the supernatants and cell lysates was detected as a low molecular weight form corresponding to decorin with approximately 40 kDa [25] (Figure 2). This product was observed in ADAM9^−/−^ and control fibroblast supernatants. ADAM9 deficient fibroblasts compared to WT controls significantly increased expression of collagen type I (Figure 2 right panel). That was also detected when ADAM9 knockout fibroblasts were stimulated with B16F1 conditioned medium (B16F1 c.m.) as compared to fibroblasts WT cultures (Figure 2C). Interestingly, a low molecular weight collagen type I fragment of about 70 KDa was observed in both fibroblast supernatants and was reduced in ADAM9 deficient fibroblast supernatants (Figure 2A). Both decorin and collagen type I, were not detected in lysates and supernatants by B16F1 melanoma cells (Figure 2C). To identify whether the difference in ECM secretion by untreated fibroblasts was due to de novo synthesis, transcripts levels of decorin and collagen type I, were analyzed by real-time RT-PCR. The relative expression ratio of decorin transcripts in ADAM9^−/−^ versus WT fibroblasts was not significantly changed in monocultures (Figure 2B). Comparable, relative transcript expression of collagen type I was higher, but not significant, in WT as compared to ADAM9^−/−^ fibroblasts monolayer (Figure 2B).

### 3.3. Impact of Collagen Type I Cleavage by ADAM9

As above described, the reduced amount of the low molecular weight fragment of collagen type I observed in supernatants from ADAM9^−/−^ fibroblasts suggests that ADAM9 may be directly involved in the cleavage and turnover of collagen type I.

**Figure 3:**
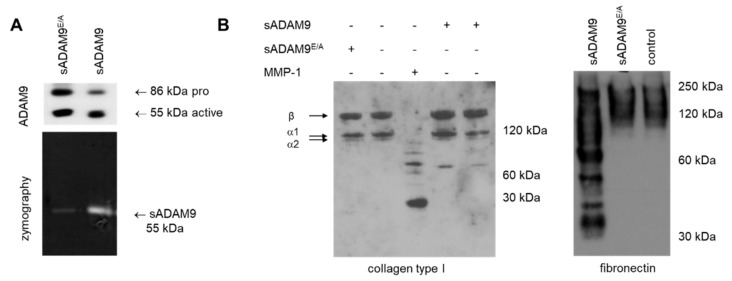
Collagen type I is a substrate of recombinant ADAM9 (**A**) Upper insert, detection of purified ADAM9 by western blot using anti-ADAM9 antibodies. In the lower panel, gelatin zymography analysis shows the high gelatinolytic activity of the catalytic active 55 kDa (sADAM9) and low of the catalytically inactive ADAM9 (sADAM9E/A). (**B**) Enzymatic processing of fibronectin or collagen type I by recombinant ADAM9s as assessed by immunoblotting. 500 ng of recombinant collagen type I or fibronectin were incubated alone (control, −) or without (−) and with one µg of recombinant sADAM9 or sADAM9E/A (+) for 16 h in substrate buffer. Incubation of collagen with 500 ng MMP-1 used as a positive control. Molecular weight markers are on the right.

To address this issue, we produced recombinant soluble ADAM9 (referred to as sADAM9) as active or catalytically inactive protein. The protein was inactivated by mutating, using site-directed mutagenesis, the glutamic acid in the zinc-binding motif of the catalytic domain to alanine (sADAM9^E/A^) [26]. The mutation was verified by sequencing. sADAM9 was purified from the COS7 culture supernatant by metal affinity chromatography and detected by western blot using antibodies against ADAM9 (Figure 3A, upper panel). We identified two bands of 86KDa and 55KDa, the first corresponding to the unprocessed inactive form containing the prodomain and the second, the active protein lacking the prodomain. That was confirmed by inhibition assays using a dec-RVKR-cmk furin inhibitor, which inhibits the processing of the prodomain [17] (Supplementary Figure S2). The catalytic activity of sADAM9 was evaluated by gelatin zymography. Processed active sADAM9 was detected as a gelatinolytic band of about 55 KDa (Figure 3A, lower panel). A minor molecular weight protein was recognized for the proteolytic inactive recombinant protein (sADAM9^E/A^), probably indicating that the mutation did not lead to 100% efficient proteolytic activity inhibition. However, in enzymatic assay with recombinant fibronectin, a known substrate of ADAM9 [10] sADAM9^E/A^ did not display any proteolytic activity, only catalytically active sADAM9 was able to cleave fibronectin (Figure 3B). To investigate if the recombinant sADAM9 can cleave collagen type I, we incubated purified sADAM9 with purified collagen type I and analyzed the resulting fragments by western blot. We could detect a proteolytic fragment of approximately 70 KDa (Figure 3B), and although this cleavage product was absent when we used the mutated inactive enzyme, the overall amount of collagen type I was not significantly altered. This 70KDa molecular weight form was further identified by peptide mass fingerprint analysis of tryptic fragments as a part of the collagen alpha 1 (I) chain. The identified four pieces of collagen were located in the middle of the molecule (aa 10–1453, MW137,9) corresponding to aa 257-267, 771-785, 983-1003, and 1073-1082. Thus, we cannot clearly state whether it is a C or N terminal fragment.

**Figure 4:**
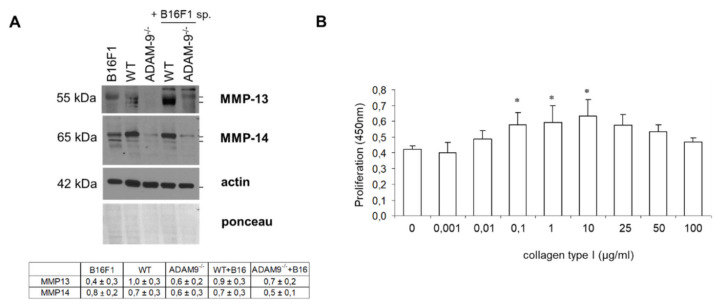
Deletion of ADAM9 leads to reduced expression of collagenolytic enzymes and thereby enhanced tumor cell proliferation. (**A**) MMP-13 and MMP-14 were analyzed by immunoblot in fibroblast supernatants (normalized to amounts of protein lysates) and lysates, respectively. Both ponceau staining of the membrane and actin immunoblot were used as the loading controls. Underneath, the quantification of MMP13 and 14 is shown (*n* = 5–9 independent fibroblasts isolation each genotype). (**B**) The proliferation of B16F1 melanoma cells was assessed in the presence of increasing concentrations of purified collagen type I by BrdU incorporation. The graph represents the mean ± SD. *p* values were calculated for cell proliferation in the presence of collagen type I compared to serum-free untreated control. * *p* < 0.05.

Collagen type I protein, but not transcripts, were altered in ADAM9 deficient fibroblasts. Furthermore, ADAM9 does not degrade collagen but only releases a fragment of the molecule. We analyzed whether altered expression of well-known collagenolytic enzymes such as MMP-13 and MMP-14 [27] may account for the accumulation of collagen in the extracellular environment. Interestingly, ADAM9 deficient fibroblasts express tendentially reduced, but not significantly (Figure 4A right Table), MMP13, and MMP14 protein amount, as compared to WT cell supernatants or lysates, respectively (Figure 4A). The latter may lead to the accumulation of collagen type I by reducing collagen degradation.

The ECM can, among other things, regulate cell proliferation [28]. Thus, we asked whether altered collagen type I expression at the melanoma-stroma border influenced melanoma cell proliferation. To this end, we analyzed the proliferation of melanoma cells in vitro on increasing concentrations of collagen type I. We could detect a biphasic proliferative response of B16F1 cells, with proliferation being increased at lower amounts of collagen and reaching a maximum at 10 µg/mL. At higher collagen concentrations, proliferation was steadily reduced (Figure 4B). This result suggests that collagen concentration induce a bell-like effect on melanoma cell proliferation.

## 4. Discussion

Melanoma progression is a stepwise process requiring the activity of different proteolytic enzymes that mediate degradation of the basement membrane and extracellular matrices, shedding of adhesion molecules, the release of growth factors, and receptors thereby playing a role in the progression of tumor cells [29,30]. Among these enzymes, ADAM9 was up-regulated in human melanoma, but its function in this context was unclear [8,31]. We have recently shown that grafted melanoma cells in the skin of mice lacking ADAM9 generated larger tumors because of increased growth of melanoma cells in vivo and confirmed by increased proliferation of melanoma cells in vitro [15]. This process was mediated by increased stromal secretion of TIMP-1 and altered proteolytic release of TNF/TNFR.

In vitro studies using recombinant proteins or overexpression, systems have highlighted the proteolytic function of ADAM9 towards matrix proteins including fibronectin (Fn1), laminin and gelatin [10,21]. In the present study, we sought to investigate the activity of ADAM9 as a peritumoral protease in the modification of the ECM in mice in vivo using proteomic analysis and in vitro in isolated dermal fibroblasts.

This analysis was performed using micro-dissected peritumoral areas of tumors grown after three days from grafting, a time point at which we previously could detect the first differences in tumor growth in ADAM9 deficient mice as compared to control mice [15]. By mass spectrometric analysis, we could detect significant differences in the expression of components as decorin, collagen type I, and collagen type V. In contrast the differences were not detected in healthy skin from these mice. The simultaneous up-regulation of both collagen type I and V is somehow not surprising as collagen type V has been often associated with collagen type I and involved in collagen fibrillogenesis [32]. The small proteoglycan decorin also binds collagen type I fibrils [33]. Thus, an increase in those three components might have occurred to generate supramolecular structures. Increased synthesis of collagen type I and other matrix components by stromal cells was reported in skin cancer [23,34]. However, in contrast to carcinomas, melanoma does not induce a robust stromal response with respect to collagen expression and fibroblast activation at the invasive front of invasive melanoma [35]. Collagen is highly expressed around tumors facing the papillary dermis, but not in deeply invading tumors. That is, in contrast, to high and widespread collagen type I expression in squamous cell carcinoma of the skin [36]. Functionally, collagen type I in melanoma was shown to reduce drug-induced apoptosis [37]. In addition, Henriet et al. (2000) demonstrated that increased proliferation of melanoma cells in contact with collagen was dependent on the organization of collagen fibrils [38]. In agreement with these studies, B16F1 cell proliferation in vivo in tumors from ADAM9 deficient mice exhibited higher proliferation at the tumor periphery, in close vicinity to areas with high collagen density as compared to tumors from WT control mice [15]. The pro-proliferative effect of collagen type I is, however, unclear. Huh and colleagues reported that collagen type I-rich ECM might be tumor suppressive [39]. In our investigation, we corroborated both of the results by showing that in vitro B16F1 melanoma cell proliferation was augmented with increasing amounts of collagen type I up to 10 µg/mL in a dose-dependent manner. However, a further increase in the amount of collagen leads to inhibition of cell proliferation, indicating a biphasic effect. Thus, it is likely that, in vivo, alterations in the collagen amount, density, conformation, and localization determine melanoma progression over time.

In the skin, fibroblasts are the significant cell type that synthesizes collagen type I and other ECM components [40], and these cells, primarily via the expression of MMP-14, are responsible for collagen remodeling in homeostasis [41]. In vitro, our analysis of fibroblasts grown as monolayers and stimulated with murine melanoma cell-conditioned medium demonstrated an increase in collagen type I secretion. As this increase was detected in fibroblasts monocultures, it indicates that enhanced matrix secretion results from the ablation of ADAM9 rather than cross-communication between melanoma cells and fibroblasts. No changes were detected in other ECM proteins investigated in fibroblasts (collagen type XIV and fibronectin), thus suggesting that in vivo different cell types present in the tumor microenvironment, for example, inflammatory cells in the peritumoral areas may be involved in the direct/indirect regulation of the expression of these proteins.

In agreement with this, also increase in decorin in vivo may result from the activity of other cell types present in the peritumoral areas, for instance, endothelial cells [42]. Since decorin is involved in collagen binding and fibrillogenesis [43,44], its increased expression in vivo may be necessary to bind collagen, sequestrate growth factors, and assemble new collagen fibril bundles. Interestingly, our in vitro studies indicated that increased collagen type I secretion by fibroblasts was not the result of enhanced transcriptional regulation, but likely the result of altered post-transcriptional events. We observed a significant reduction of a low molecular weight collagen alpha 1(I) chain fragment of about 70 kDa in the supernatants of ADAM9 deficient fibroblasts.

Using the functional, active ADAM9 recombinant or proteolytically inactive mutant and monomeric/triple helical collagen type I as a substrate, we could demonstrate in vitro that ADAM9 was directly responsible for the proteolytic release of the 70 kDa fragment of the Collagen alpha 1 (I) chain. However, being the total amount of collagen type I not reduced in the presence of recombinant ADAM9, we conclude that ADAM9 does not degrade collagen. If the release of the 70 kDa collagen fragment results in destabilization of collagen molecules or the induction of other cellular activities is not known and will be a matter of future investigations.

Collagenolytic enzymes as MMP-13 and MMP-14 are involved in melanoma development where they mediate the vascularization of tumors or tumor cell migration during tissue invasion [45,46]. A tendential reduction of the collagenases MMP-13 and MMP14 was found in ADAM9 deficient fibroblasts. Thus, this reduction would suggest that increase in collagen may occur by reduced collagenolysis. In a previous analysis, we showed that ADAM9 deleted fibroblasts secrete a high amount of TIMP-1 in the peritumoral area of the ADAM9 deficient animals [15]. TIMP1 was efficient in the inhibition of a variety of enzymes, including collagenases [47]. Thus, it is possible that accumulation of collagen in peritumoral areas in ADAM9^−/−^ mice is the result of reduced and/or inhibited collagenolytic activities.

In summary, we have shown that the expression of ECM components during melanoma development is altered in the absence of ADAM9. We observed increased collagen type I at the tumor–stroma border of melanomas from ADAM9^−/−^ animals, which results from altered fibroblast activities. Hence, we provide evidence that ADAM9 can release a fragment of collagen type I, but not degrade it, and that indirectly, ADAM9 contributes to the regulation of collagen type I expression in peritumoral areas, thereby providing modulation of B16F1 cell proliferation during melanoma development.

## 5. Conclusions

In summary, we report the differential expression of several matrix proteins, including decorin, collagen type XIV, fibronectin, and collagen type I in the absence of stromal-derived ADAM9. We identified increased collagen type I as a result of ADAM9 deletion in the tumor microenvironment. Further, we show that collagen type I density influences the proliferation of B16F1 melanoma cells in vitro.

## Supplementary Materials

The following are available online at https://www.mdpi.com/2218-273X/10/9/1290/s1, Table S1. Differential expression of enzymes detected in the absence of ADAM9. Fold change of proteins analyzed by mass spectrometry in ADAM9^−/−^ tumor stroma surrounding compared to WT. Positive values indicate up-regulation and negative values indicate down-regulation of proteins. Figure S1. ECM proteins in healthy skin of WT and ADAM9^−/−^ animals. Immunofluorescence analysis of collagen type I, collagen type XIV, decorin, and fibronectin (green, nuclei: blue). As a negative control, sections were incubated in a blocking buffer without the primary antibody. Representative pictures are shown (WT *n* = 5; ADAM9^−/−^ *n* = 5). On the right, the relative expressions of the proteins analyzed were used to calculate the fold change of proteins in ADAM9^−/−^ as compared to WT healthy skin. Figure S2. Detection of soluble ADAM9 in supernatant from COS-7 cells transfected with soluble ADAM-9 plasmid, with or without treatment with CMK.
