# Supplementary material for: Loss of ADAM9 Leads to Modifications of the Extracellular Matrix Modulating Tumor Growth
Source: Biomolecules. 2020 Sep 7;10(9):1290. doi: 10.3390/biom10091290 (PMC7564588; doi:10.3390/biom10091290)

Table S1

**Enzymes**	**MW**	**fold change ADAM-9 $^{-/-}$ vs WT tumor**
Fatty acid synthase	272 kDa	-8,16
Long-chain-fatty-acid--CoA ligase 1	78 kDa	-2,67
triosephosphate isomerase 1	32 kDa	4,39
Pyruvate kinase isozymes R/L	62 kDa	2,20
trypsin 4	26 kDa	-2,73
Mast cell protease 4	27 kDa	-2,72
Peroxiredoxin-1	22 kDa	2,20
Peroxiredoxin-4	31 kDa	2,20
Creatine kinase M-type	43 kDa	-3,00

Figure S1

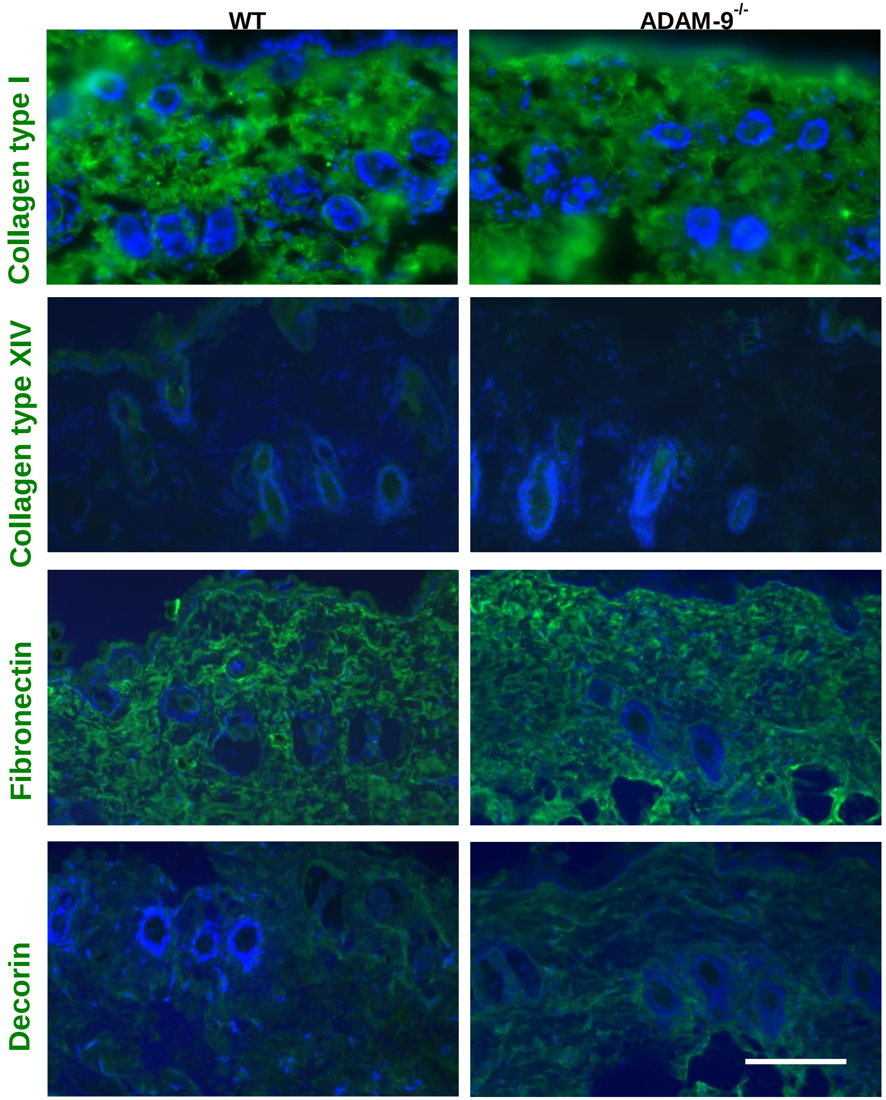

ECM Proteins	fold Adam-9 $^{-/-}$/WT skin
Isoform 1 of Collagen alpha-1(I) chain	1
Collagen alpha-2(I) chain	1
Collagen alpha-1(III) chain	1
Collagen alpha-2(V) chain	1,5
Isoform 1 of Collagen alpha-1(XIV) chain	0
Fibronectin	0
Decorin	1,5

Figure S2

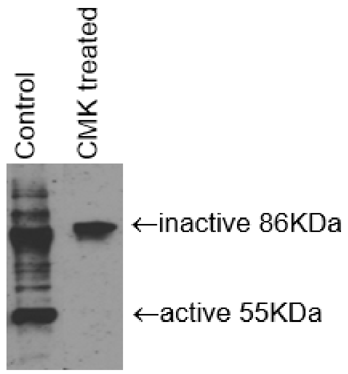

Supplement: Supplementary file 1 [file biomolecules-10-01290-s001.pdf]
